# The contribution of general practice based research to the development of national policy: case studies from Ireland and Australia

**DOI:** 10.1186/1743-8462-3-4

**Published:** 2006-05-11

**Authors:** JE Pirkis, GA Blashki, AW Murphy, IB Hickie, L Ciechomski

**Affiliations:** 1 Program Evaluation Unit, School of Population Health, The University of Melbourne, Melbourne, Australia; 2 Health Services Research Department, Institute of Psychiatry, King's College London, London, UK; 3 Department of General Practice, National University of Ireland, Galway, Ireland; 4 Brain and Mind Research Institute, University of Sydney, Sydney, Australia; 5 Department of General Practice, School of Primary Health Care, Monash University, Melbourne, Australia

## Abstract

**Background:**

This paper aims to describe the influence of general practice based research on the development of two specific policy initiatives, namely the Heartwatch Programme in Ireland and the Better Outcomes in Mental Health Care (BOiMHC) program in Australia. A case study approach was used to explore the extent to which relevant general practice based research shaped these initiatives.

**Results:**

In both case studies, a range of factors beyond general practice based research shaped the initiative in question, including political will, the involvement of stakeholders (including key opinion leaders), and the historical context. Nonetheless, the research played an important role, and was not merely put to 'symbolic use' to support a position that had already been reached independently. Rather, both case studies provide examples of 'instrumental use': in the case of Heartwatch, the research was considered early in the piece; in the case of the BOiMHC program, it had a specific impact on the detail of the components of the initiative.

**Conclusion:**

General practice based research can influence policy-making and planning processes by strengthening the foundation of evidence upon which they draw. This influence will not occur in a vacuum, however, and general practice researchers can maximise the likelihood of their work being 'picked up' in policy if they consider the principles underpinning knowledge transfer.

## Introduction

Over the years, considerable attention has been paid to the extent to which health services research findings influence health policy directions. As far back as the 1970s, various authors began to consider how research findings could be used. Weiss, for example, identified taxonomy of types of research use that comprised instrumental, conceptual and symbolic uses. 'Instrumental use' occurs when research findings are acted on in specific and direct ways, such as when evaluation results are used to reshape a given health program. 'Conceptual use' is more indirect, and relates to building the overall knowledge base in a given area. 'Symbolic use' involves drawing on research to justify a position or action that has already been taken for another reason [1].

More recently, various authors have progressed work in this area, considering theoretical and practical explanations for the extent to which research findings are (or are not) taken up in policy decisions, and the reasons for their use (or non-use). Innvaer et al [2] conducted a systematic review of 24 studies in which policy-makers' views were sought regarding perceived facilitators and barriers to using research evidence for policy decisions. They identified seven facilitators (personal contact between researchers and policy-makers; timeliness and relevance of the research; research that included a summary with clear recommendations; good quality research; research that confirmed current policy or endorsed self-interest; community pressure or client demand for research; and research that included effectiveness data). They also identified six barriers (absence of personal contact between researchers and policy-makers; lack of timeliness or relevance of research; mutual mistrust, including perceived political naivety of scientists and scientific naivety of policy-makers; power and budget struggles; poor quality of research; and political instability or high turnover of policy-makers). Lavis et al [3] expanded on this work by updating the review, interviewing policy-makers, and reviewing relevant research websites that included policy-makers among their target audience. They focused specifically on research evidence presented in systematic reviews, and identified similar facilitators and enablers to those of Innvaer et al [2].

Lavis and colleagues have discussed the relative impacts of 'producer-push' approaches (where producers of research actively push research knowledge out to users of research), 'user-pull' approaches (where users of research actively pull in research when faced with a decision that they believe could be informed by research knowledge) and 'knowledge exchange' approaches (where producers and users of research are jointly responsible for transferring and facilitating the uptake of research knowledge, and elements of 'producer push' and 'user pull' approaches occur) [4,5]. Lomas [6] has also discussed the notion of 'linkage and exchange', espousing the view that routine and ongoing involvement of policy-makers in the activities of research organisations (e.g., setting priorities, funding programs, conducting research and communicating findings) will improve the likelihood of research influencing policy.

It is fair to say that, to date, most of the work that has examined the influence of health services research on policy has concerned itself with the secondary and tertiary health sectors, and has paid little heed to the primary care or general practice sector. There may be a number of reasons for this, including the relative costliness of the former two sectors (and hence a drive for policies that maximise their efficiency), and the fact that general practice based research is a comparatively young enterprise and has therefore had less opportunity to have an impact on policy.

It is also fair to say that the situation seems to be changing. Internationally, there is an increasing emphasis on the importance of general practitioners as lynchpins in the health system. As generalists in the community, general practitioners have a first contact, longitudinal and comprehensive perspective of patients' complaints, and are therefore recognised as crucial stakeholders in the delivery of agreed national policy. In addition, there has been a growing strengthening of general practice based research worldwide, with, for example, a burgeoning of academic departments of general practice, improvements in funding opportunities for general practice based research, increases in the numbers of general practitioners who are combining clinical and research careers, and expansions in the forums through which general practice based research can be showcased (e.g., peer-reviewed journals, conferences). Together, these changes create opportunities for examining the extent to which evidence from general practice based research has been applied to new policy development.

The current paper describes the authors' experiences in Ireland and Australia with regard to the influence of general practice based research on the development of specific cardiovascular and mental health policy initiatives, respectively. Ireland and Australia were chosen on the basis that they have in common health systems that are funded through a complex and evolving mixture of central taxation, private insurance, social insurance and out-of-pocket payments [7,8]. Both countries also share a strong central policy-making approach [7,8]. The strength of their primary care systems is also similar, as reflected in their scores on the Starfield scoring system, which both fall into the intermediate range [9-11]. The Starfield score is derived by rating health system and practice characteristics related to primary care, including: the type of system; financing; type of primary care practitioners; ratio of primary care physicians to specialists and their relative earnings; patient lists; requirements for 24 hour coverage; and strength of academic departments of family medicine. Each item is assigned a score depending on the relative development of each characteristic. The unweighted scores are then averaged to derive a 'primary care score', with higher scores indicating stronger primary care.

## Method

A 'two-case' exploratory case study approach was used, following Yin [12]. The two cases descriptively examined the following research question: 'To what extent did relevant general practice based research shape the given initiative, and what other competing influences on policy were present?'

The authors drew on three data sources that are commonly used in case study designs, namely documentation (in this case, relevant research and policy documentation), observation (including participant-observation), and supplementary information gathered by informal, ad hoc interviews with selected key informants [12].

Case descriptions were developed for each of the two cases, which involved outlining the processes involved in each of the two initiatives and considering the relative impact of research in the causal pathways of development and implementation [12].

## Results

### The Irish experience

In the late 1990s, the Department of General Practice at the National University of Ireland, Galway, provided the first baseline data on the provision of secondary cardiac care in Irish general practice [13]. Data were gathered on 1,611 patients with established heart disease from a random sample of 35 general practices. The provision of secondary cardiac care within Irish general practice was shown to be sub-optimal and comparable to other similar national studies [14,15].

At around the same time, the Department of Health and Children published 'Building Healthier Hearts: A National Cardiovascular Strategy' [16] and established a national Advisory Forum on which AWM was the sole general practitioner member. To develop and prioritise strategy, a primary care sub-committee was formed which was chaired by AWM. This sub-committee included representatives of the Irish College of General Practitioners, local health authorities, the Irish Heart Foundation and the Department of Health and Children. It was agreed by both the sub-committee and Advisory Forum that, of the 92 recommendations relating to primary care, secondary prevention of cardiac disease was the priority for primary care. AWM then chaired an implementation group which was charged with the task of agreeing the principles of secondary prevention implementation. This was an arduous task, with 'ownership' of practice data – between general practice and local health authority – proving particularly problematic. A successful outcome to this process, of an agreed national programme, was only achieved through sustained collaboration between all the major stakeholders. The programme became known as Heartwatch, and is described in detail in Table 1.

**Table 1:** Heartwatch (Ireland)

Heartwatch involves the evaluation of the first phase of a structured programme of secondary prevention of cardiovascular disease in general practice in Ireland. The overall aim of the programme is to reduce morbidity and mortality due to cardiovascular disease. Heartwatch is implementing the guidelines outlined by the Second Joint Task Force of European and other Societies on Coronary Prevention ('Prevention of Coronary Disease in Clinical Practice 1998') for the first time in the context of a national programme.
The Heartwatch Programme was agreed by the Department of Health and Children, the Health Boards and the Irish College of General Practitioners in collaboration with the Irish Heart Foundation, and is the culmination of several years of preparatory work. A National Programme Centre, Independent National Data Centre and regional infrastructures and processes have been established to implement and manage the Heartwatch Programme. The budget for 2004, excluding accruals is three million euros. The initial implementation phase focuses on secondary prevention amongst those with significant proven cardiovascular disease (e.g., history of acute myocardial infarction, coronary artery bypass surgery or PTCA).
The programme targets 20% of Irish general practitioners and provides a protocol for the continuing care of eligible patients including a schedule of four visits per annum initially, and details of the risk factors to be measured with targets levels of control to be achieved.
An explicit schedule of payments to general practitioners was agreed; care may be provided by a practice nurse. Payments are issued on a monthly basis to practitioners based on information supplied by the INDC to the GMS Payments Board.
There are now in excess of 11,400 patients in the programme and over 50,000 continuing care visits have taken place.
Heartwatch is significant in that it represents the first attempt to provide structured chronic disease management within Irish general practice to all patients irrespective of patient income.

*Source: *Adapted from Irish College of General Practitioners [36]

During the policy-development and planning activities that underpinned Heartwatch, AWM championed the use of the above research data on the current status of secondary prevention in general practice. As a result, the research findings informed strategies in the following areas:

• Confirming and characterising the 'treatment gap' between recommended secondary cardiac care and current practice by general practitioners;

• Developing a template for identifying patients with established heart disease in a general practice system without universal patient registration;

• Quantifying eligible numbers of patients per individual practice; and

• Costing of potential budgetary implications according to varying funding mechanisms.

In the case of Heartwatch, relevant and applicable general practice based research 'pushed' by a member of the policy formulation team was considered to have played a positive and influential role, particularly in the early stages. The research in question identified a treatment gap which, given appropriate resources, could be relatively easily bridged to achieve a significant health gain. Having said this, there were certainly other competing influences on the policy development of Heartwatch, including the sometimes-conflicting, sometimes-overlapping interests of stakeholders that emerged as the process progressed. These interests meant that many stakeholders did not consider the research findings in their decision-making. Arguably, without the research, the policy development process may have concluded with the same outcome.

### The Australian experience

Over the last decade in Australia, there has been an increasing emphasis on the role of primary care providers (particularly general practitioners) in the delivery of mental health care. Two overarching policy frameworks have guided this movement: the National Mental Health Strategy, which stresses the need for mutually supportive partnerships between the primary and secondary care sectors; and the General Practice Strategy which argues that health outcomes in general could be improved through more appropriate primary care services [17].

Within this context, the Australian Government provided funding in the 2001–02 Budget for the Better Outcomes in Mental Health Care (BOiMHC) program (see Table 2). The Federal Health Minister formulated the General Practice Memorandum of Understanding (GP MoU) Group to develop the details of the program, which in turn sought policy and planning advice from a national committee known as the Committee for Incentives in Mental Health (CIMH). CIMH comprised representation from the Australian Divisions of General Practice (ADGP), beyondblue: the national depression initiative, the Mental Health Council of Australia (MHCA), the Royal Australian College of General Practitioners (RACGP), the Royal Australian and New Zealand College of Psychiatrists (RANZCP), the Australian Psychological Society (APS), the Rural Doctors' Association of Australia (RDAA), the Australian Medical Association (AMA), and the Department of Health and Aged Care (DHAC). CIMH established three task groups to provide it with advice on the development of specific areas of the program: the Education and Training Task Group, the Medicare Benefits Scheme (MBS)/Incentives Task Group, and the Allied Health Task Group. Each Task Group had representation from all of the organisations represented on CIMH. The Task Groups advised CIMH, which made recommendations to the GP MoU Group, which put them to the Minister for consideration and formal ratification [17]. IBH co-chaired CIMH and IBH and GAB both sat on the Education and Training Task Group.

**Table 2:** Better Outcomes in Mental Health Care (Australia)

The 2001–2002 Federal budget initiative Better Outcomes in Mental Health Care (BOiMHC) seeks to improve the mental health care available to Australians. It has five interconnected components, each of which is described below:
1. *Education and training for general practitioners: *Through this component, general practitioners can participate in Familiarisation Training which introduces them to the Better Outcomes in Mental Health Care program (2 hours), then Level 1 Training which equips them to perform the 3-step mental health process (6 hours), described below and then Level 2 Training which provides them with the skills necessary to undertake focused psychological strategies (20 hours), also described below. Level 1 Training is a prerequisite for participation in the other components of the initiative. An RACGP sub-committee called the General Practice Mental Health Standards Collaboration oversees this component.
2. *The 3 Step Mental Health Process: *This component provides a framework for general practitioners to manage mental health problems, and includes an assessment (Step 1), preparation of a mental health plan (Step 2) and a review (Step 3). General practitioners who have completed Level 1 Training can access a Service Incentive Payment from Medicare Australia (the body responsible for administering Medicare) for providing the 3-step process.
3. *Focused Psychological Strategies: *This component promotes evidence-based focused psychological strategies, namely psycho-education, cognitive behavioural therapy and interpersonal therapy. These strategies are normally delivered by general practitioners in planned sessions, each lasting a minimum of 30 minutes. General practitioners who have completed Level 2 Training can bill Medicare Australia against specific Medicare item numbers which have been created to recompense them for their time in delivering focused psychological strategies.
4. *Access to Allied Psychological Services: *Through this component, general practitioners who have completed Level 1 Training are able to refer consumers to allied health professionals (e.g., psychologists, social workers) for the same focused psychological strategies described above. The allied health professionals are contracted to or employed by Divisions of General Practice through Access to Allied Psychological Services projects.
5. *Access to Psychiatrist Support: *This component enables psychiatrists to be reimbursed for participating in case conferences with general practitioners and others, and provides access to patient management advice to general practitioners from psychiatrists through a GP Psych Support service.
By the beginning of 2006, 4,467 general practitioners had completed Level 1 Training, and 902 had completed Level 2 Training. General practitioners had completed 48,736 3 Step Mental Health Processes and delivered 59,996 sessions of Focused Psychological Strategies. One hundred and eight Access to Allied Psychological Services projects had been funded, enabling 2,980 GPs to refer 26,444 patients to 1,040 allied health professionals.

*Source: *Adapted from Pirkis et al [37]

In formulating the details of their advice and recommendations, CIMH and the Task Groups were informed by relevant research. Not all of this research could be described as general practice based. The rationale for the program was established with reference to a number of relevant community-based studies conducted by health services researchers, epidemiologists and health economists. Together, the National Survey of Mental Health and Wellbeing, the Australian Burden of Disease Study and the Australian Mental Health Literacy Survey demonstrated that depression and anxiety were significant public health problems, and that general practitioners were trusted by people with these problems and consequently provided the majority of their care [18-20].

Beyond this, however, much of the detail of the program was founded on the results of general practice based studies. For example, CIMH drew on the findings of the Australian SPHERE study of 46,515 general practice consultations, which demonstrated that general practitioners' diagnostic and management skills were sub-optimal [21,22], and international general practice based research examining the types of training that have been shown to improve these skills [23-26]. As a consequence, the BOiMHC program included accredited mental health training as a mandatory requirement for general practitioners. Likewise, CIMH relied on general practice based research evidence in developing an argument that general practitioners ought to undertake systematic assessment, planning and follow-up as part of the routine management of patients with mental health problems [27-29]. Models of care identified in research studies as likely to achieve good results informed the conceptualisation of what became the Focused Psychological Strategies [30,31]. Similar recourse to general practice based research literature guided the development of the other components of the BOiMHC program [32,33].

As with Heartwatch, a range of other influences also shaped the BOiMHC program. There was strong political will, as the Health Minister was committed to the implementation of reforms in both the mental health and primary care sectors, and the DHAC was intent on improving health outcomes across the board. The program was 'driven' by key opinion leaders [17]. There was unprecedented collaboration between major advocacy and professional groups, and the Task Groups sought the input of their respective organisations and/or constituents prior to agreeing to a particular position [17]. Historical factors, including Australia's mental health policy context that was fostering community based care, also set the scene for change. Notwithstanding this, the detail of the program described in Table 2 owes much to general practice based research.

## Discussion

The current paper provides two case studies of general practice based research being used to guide decision-making about key, national health initiatives. We acknowledge the limitations of a case study approach, recognising issues of generalisability, potential observer bias, subjectivity and possible differences of interpretation. However, we believe that the case studies provide a useful starting point for considering the impact that general practice based research can have on policy.

There is a vast theoretical literature on the causal influences on public policy [34], but it is beyond the scope of this paper to review this literature here. What can be said is that both case studies acknowledge that a range of factors beyond general practice based research shaped the initiative in question, including political will, the involvement of stakeholders (including key opinion leaders), and the historical context. Nonetheless, the research played an important role, and was not merely put to 'symbolic use' to support a position that had already been reached independently. Rather, both case studies provide examples of research evidence being put to 'instrumental use' in shaping the given initiative.

Lavis et al [35] have argued that professional and technical 'content driven' decisions may be more amenable to the influence of research than broader decisions concerned with assigning jurisdictional responsibilities, and there is some evidence of this occurring in both case studies. In both instances, national policy decisions were made to develop and implement the given program, and then the professional and technical details were 'filled in', with relatively greater recourse to the research evidence in the latter part of the process. In the case of Heartwatch, the research shaped the overall initiative; in the case of the BOiMHC program, the research had a specific impact on the detail of the components of the initiative.

Both case studies can arguably be best described as exemplifying 'knowledge exchange' approaches in terms of knowledge transfer, with shared responsibility for transferring and facilitating the uptake of research knowledge, mutual respect for the knowledge that different parties brought to the table, and development of jointly 'owned' knowledge [5]. They involved a 'user pull' element, where decision-makers (either individuals or committees) insisted on referring to research evidence in the planning process. However, the decision-makers were unusual, in that some wore research hats as well, which may have facilitated the knowledge transfer process by increasing their awareness of the importance of working with a sound evidence base and their understanding of how to interpret research findings. Indeed, AWM, IBH and GAB had all been involved in at least some of the research studies that were being drawn upon, thereby introducing a 'producer-push' element. This coincidence of roles overcame the commonly-cited barrier of absence of personal contact between researchers and policy-makers, and may have been the key contributor to successful knowledge transfer. It is interesting to speculate whether, had this overlap in roles not existed, different approaches would have been required (e.g., whether 'knowledge brokers' might have been required to translate the research into actionable, policy-relevant messages).

The case studies suggest that general practice based research can contribute to decision-making about initiatives that will affect general practitioners 'on the ground' and have flow-on effects for their patients. The corollary of this is that general practice researchers should be well supported (e.g., through appropriate training and the provision of 'protected time'), and should be encouraged to consider features of the process that may facilitate or impede the influence of their work on policy (e.g., the nature of the research, the clarity or 'actionability' of the research messages, their own 'knowledge transfer' skills, and the 'knowledge uptake' skills of the decision-makers). General practice researchers should also be encouraged to take up positions on key decision-making bodies, in recognition of the fact that they may be able to facilitate 'knowledge exchange' approaches to maximise the influence of their own work and the work of others on decision-making.

To conclude, general practice based research can contribute to policy-making and planning processes by strengthening the foundation of evidence upon which they draw. This influence will not occur in a vacuum, however, and general practice researchers (like other health services researchers) can maximise the likelihood of their work being 'picked up' in policy if they consider the principles underpinning knowledge transfer.

## Declaration of competing interests

The author(s) declare that they have no competing interests.

## Authors' contributions

JP, GB and AM conceptualised the idea for the paper, developed the case studies, and took the lead on preparing the manuscript. IH and LC contributed substantially to revised drafts. All authors read and approved the final manuscript.
